# Electromagnetic Acoustic Detection of Pipe Defects Hidden above T-Type Support Structures with Circumferential Shear Horizontal Guided Wave

**DOI:** 10.3390/mi15040550

**Published:** 2024-04-20

**Authors:** Xingjun Zhang, Jinjie Zhou, Yang Hu, Yao Liu, Xingquan Shen

**Affiliations:** 1 School of Mechanical Engineering, North University of China, Taiyuan 030051, China; 13015482673@163.com (X.Z.); hy15536307062@163.com (Y.H.); liuyao@nuc.edu.cn (Y.L.); jdztczjj1@126.com (X.S.); 2 Shanxi Key Laboratory of Intelligent Equipment Technology in Harsh Environment, Taiyuan 030051, China

**Keywords:** EMAT, shear horizontal, pipeline support structure, waveform subtraction, defect detection

## Abstract

When pipe defects are generated above the T-type support structure location, it is difficult to distinguish the reflection signals caused by the weld bead at the support structure from the reflection echoes of pipe defects. Therefore, in order to effectively detect pipe defects, a waveform subtraction method with a circumferential shear horizontal (CSH) guided wave is proposed, which is generated by an electromagnetic acoustic transducer (EMAT). First, a CSH_0_ guided wave mode with a center frequency of 500 kHz is selected to establish a three-dimensional model with and without pipe defects above the support structure. Following this, the influence of different widths of support structures on the echo signal is compared. Moreover, simulation and experimental results are used to compare the influence of different welding qualities on the detection results. Finally, the waveform subtraction method is used to process the simulation and experimental signals, and the influence of pipe defects with different lengths and depths is discussed. The results show that the non-through crack defect of 5 mm × 1 mm (length × depth) can be detected. The results show that this method can effectively detect the cracks by eliminating the influence of the weld echo, which provides a new concept for the detection of the defect above the support structure.

## 1. Introduction

In recent years, the construction of oil and gas transportation pipelines has been in full swing due to the harsh working environment of the pipelines. With the increase in use time, a variety of cracks, corrosion, and even perforation has occurred, which can led to catastrophic accidents [1]. There is an urgent need to conduct a comprehensive assessment of the damage to the pipeline regularly. To enhance the stability and reliability of pipeline transportation and to prevent the pipeline from sagging or collapsing, pipelines must be supported with columns at intervals, which results in a large number of supporting brackets. The pipeline at the support area can easily incur defects due to the dual role of internal and external corrosion. Unsupported pipeline defect detection can use ultrasound [2,3], magnetic leakage [4,5], eddy current [6,7], rays [8], and other traditional methods of detection. However, supported pipeline detection is difficult, especially when the pipeline has been damaged. Lifting the pipeline from the support and carrying out conventional detection is not only time-consuming and laborious but also dangerous. The defects in the supported pipeline area, such as cracks and corrosion defects, may lead to leakage. Therefore, there is an urgent need for a rapid non-destructive detection method for pipeline defects at the support area.

Many researchers have explored the effects of pipe supports. Khalili et al. used a piezoelectric probe to excite an A1 modal guided wave at 2 MHz to detect corrosion defects 1 mm deep in pipeline supports [9]. Shivaraj et al. used a piezoelectric transducer to excite an A1 modal guided wave at 1 MHz and detected defects with a diameter of 1.5 mm and a depth of 2 mm [10]. Piezoelectric detection has high requirements on the specimen surface, which has poor experimental repeatability. However, electromagnetic ultrasonic guided waves have the advantages of non-contact, no coupling, fast detection speed, high efficiency, long propagation distance, and small attenuation, and are widely used in practical detection.

Guided waves in pipelines can be divided into axial and circumferential guided waves according to the propagation direction, and axial guided waves in pipelines can be used for long-distance and fast detection, which has been widely studied. Wang et al. designed a 320 kHz PPM EMAT to study the propagation characteristics and defect detection capability of the T(0,1) mode guided wave in aluminum pipe bends. In terms of propagation characteristics, the energy of the guided wave is concentrated at the outer side of the bends, and due to the difference in propagation distances, the guided waves at the inner side of the bends and at the outer side of the bends are separated. In terms of defect detection capability, the T(0,1) mode guided wave has the highest sensitivity for detecting defects on the outside of the bend and the lowest sensitivity for detecting defects in the middle region of the bend [11]. Liu et al. proposed a method that uses an improved planar solenoid array coil which can effectively generate and receive the fundamental torsional modes T(0,1) in pipelines and successfully identified 1.5 mm deep circumferential groove crack defects in pipes with an outer diameter of 42 mm and a thickness of 5 mm [12]. Quiroga et al. proposed a torsional-guided-wave-based stiffness monitoring scheme for cylindrical structural braces for detecting the loss of braces’ stiffness [13].

In recent years, some scholars have proposed a detection technology based on circumferential guided waves for detecting pipeline structures. Deng et al. used a Lamb wave to detect through-holes with 1 mm diameter and 0.4 mm depth grooves in a 4 mm thickness plate [14]. Shi et al. established a SH_0_ guided wave generated by PPM EMAT to detect circumferential cracks. The effect of the geometrical parameters of the circumferential cracks on the two types of edge-reflected waves was examined. The results showed that the wave peaks of the cracks show a trend of linearly increasing with the crack’s circumferential cross-sectional area. When the circumferential range of the crack increases, the peak of the crack-trailing edge wave exhibits roughly periodic changes. The relative deviation between the estimated circumferential range and the actual range is less than 5.1% when the range of the crack circumference is not less than 10 mm. The correlation information of the two edge-reflected waves can provide an accurate and quantitative characterization of pipeline defects [15]. Zhang et al. proposed a method by which to excite CSH_0_ modes and first-order cyclic shear horizontal (CSH_1_) modes via a bi-directional SH wave piezoelectric transducer. The experiments on 11 mm-thick steel pipe showed that the method can accurately determine the depth and circumferential extent of the notch. These were performed to investigate the reflection and transmission characteristics of CSH_0_ and CSH_1_ waves from notches with different depths [16]. Hirao et al. examined natural gas pipelines with PPM EMAT and concluded that the phase shifts of the SH_0_ modes correlate with the remaining thickness based on the fact that circumferential shear waves are non-dispersive [17].

SH wave frequency dispersion is simple. When encountering obstacles and defects, it is not easy to mode shift, reducing the complexity of the received signal. The particle displacement direction of the SH_0_ wave is in-plane and less affected by the surrounding medium. Secondly, SH_0_ waves are very suitable for detecting curved structures because they can ignore the reflection problem in the propagation around the curved surface [18]. So, SH_0_ waves have the potential to detect defects across the support frame structure, applying circumferential guided waves to detect defects at the weld seams, which are highly susceptible to defects, and in the pipe support region [19]. For example, Nicholas et al. found that the support structure has less influence on the propagation of SH waves, so the experiment used EMAT to excite the SH_1_ mode at 730 kHz and successfully detected the thinning region inside the support structure [20]. Hu et al. were able to effectively detect crack defects of more than 3 mm when they utilized the lower-order CSH_0_ modes for cross-support detection of the pipeline, which provides a basis for large-area defects of the support pipeline detection studies [21].

None of the above studies have been conducted for the pipe defects hidden in the support structure, and the detection of such defects needs to pass through the welded structure at the support, so the defect signal will be hidden in the weld echo, increasing the difficulty of its detection. Further analysis and research are needed. In this paper, for the pipe defects hidden in the supports, we adopt the CSH_0_ modes with a low order of excitation for defect detection and use the waveform subtraction method to eliminate the signal interference of the weld. The rest of the paper is organized as follows: The second section introduces the propagation characteristics of SH waves and the transducer mechanism of the ferromagnetic material PPM EMAT. The third sections establishes a three-dimensional model of the existence and inexistence of pipe defects in the stent and makes a comparative analysis using a cloud image. The fourth section compares the influence of different widths of support structures on the echo signal and the influence of different weld structures on pipeline defect detection through experimentation and simulation. The fifth section analyzes and discusses the defect signals of different lengths and depths hidden in the pipe support. The sixth section summarizes the work of this paper.

## 2. Excitation Principle of PPM EMAT

The mechanism of EMAT mainly consists of Lorentz force, magnetostrictive force, and magnetization force [22]. The contribution of magnetization force is smaller compared with the other two. When a magnetic field is applied to ferromagnetic materials, the transducer mechanism becomes complicated due to the magnetization of the specimen. The transducer mechanism changes with different degrees of magnetization. In the electromagnetic ultrasonic energy conversion process of ferromagnetic materials, the magnetostrictive effect is the dominant factor in energy conversion when non-magnetically saturated. The Lorentz force plays a dominant role when magnetically saturated.

**Figure 1:**
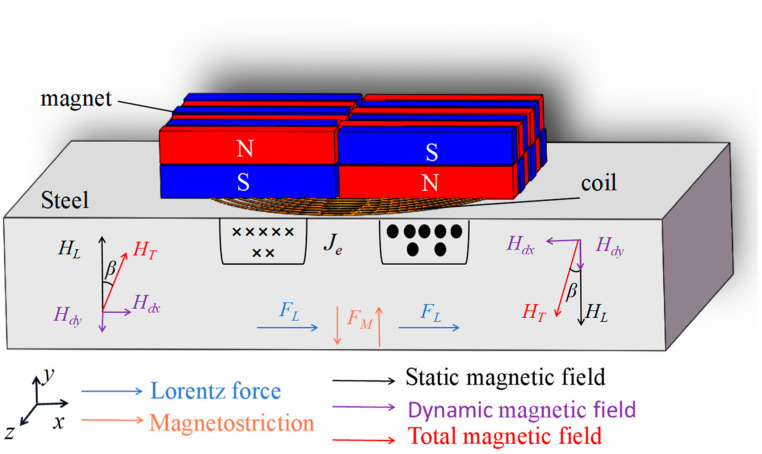
Excitation principle of PPM EMAT.

The electromagnetic ultrasonic SH guided wave sensor used in this article consists of a periodic permanent magnet and a runway-shaped coil. The specific energy transfer process of periodic permanent magnet electromagnetic ultrasonic transducers is based on Lorentz force. As shown in Figure 1, periodic permanent magnets generate a static magnetic field on the *y*-axis. The magnetic fields of adjacent permanent magnets alternate in the upper and lower directions. The current I of the runway-shaped coil is parallel to the surface of the plate in the *z*-direction. When an excitation signal is generated in the coil, a dynamic magnetic field is generated, and eddy currents of the same frequency are induced within the skin depth of the lower specimen surface. The eddy currents *Je* on both sides of the runway-shaped coil have opposite directions. The interaction among the static magnetic field, dynamic magnetic field, and eddy currents can create Lorentz forces in the *x*-axis, resulting in the vibration of particles inside the specimen. Furthermore, SH guided waves propagating in both directions along the *z*-axis are excited.

At room temperature, the bias magnetic field strength provided by the permanent magnet in PPM EMAT is much greater than the dynamic magnetic field strength, that is, *H_L_* >> *Hdx*, *Hdy*; *β* ≈ 0. Therefore, the magnetostrictive force of *F_M_* is negligible. The Lorentz force is the main ultrasonic source of SH guided wave, and the force *F* generated by EMAT can be calculated as follows:

(1) $$F=F_{L}+F_{M}\approx F_{L}.$$

**Figure 2:**
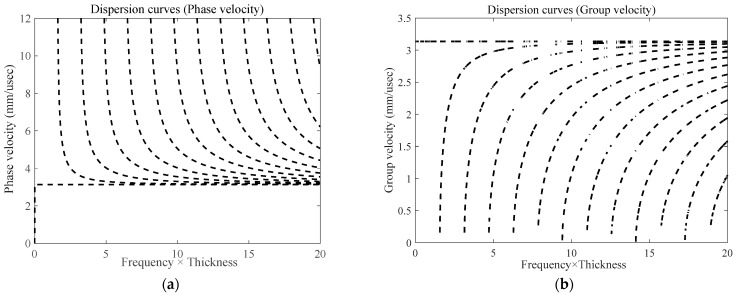
Dispersion diagram of SH guided wave: (**a**) phase velocity dispersion curve; (**b**) group velocity dispersion curve.

The dispersion curve of an approximately 3 mm-thick steel plate is shown in Figure 2. The SH wave excited by PPM EMAT has only a single SH_0_ mode at low frequency, and the wave has the advantages of no dispersion, almost independent of the wave speed and frequency thickness product, and small attenuation when detecting the wave. The wave structure is simple, or no dispersion and mode conversion will occur, which is very suitable for the needs of the defects detected at the support.

## 3. Finite Element Simulation

### 3.1. Model Building

**Figure 3:**
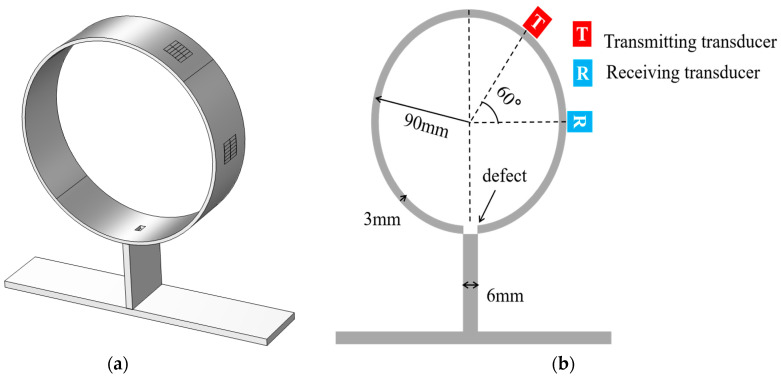
Schematic diagram of pipeline structure: (**a**) 3D models; (**b**) detection sectional drawing.

A 3D time-domain simulation model for defect detection of 3 mm-thick pipeline support is established. The transducer consists of six couples of periodic array magnets with runway-type coils. The dimensions of the periodic array magnets are 10 mm × 3 mm × 10 mm (length × width × height). A Q235 steel pipe with an outer diameter of 180 mm and a thickness of 3 mm (*ρ* = 7850 kg/m^3^; *E* = 210 GPa; *ν* = 0.30) is used as the waveguide. T-type support is formed by welding a vertical plate and a bottom plate. The size of the vertical plate is 1 m × 70 mm × 6 mm (length × width × height), and the size of the bottom plate is 1 m × 250 mm × 6 mm (length × width × height), which is used to simulate the propagation law of the guided wave at the support. To minimize interference with the detection, the surfaces on both sides of the pipeline, the ends of the support, and the bottom of the support are set up as low-reflection boundaries, and the surface of the part is smooth. However, in the experiment, there is a weld between the pipe and the support. To simulate the experiment, the density and Young’s modulus of the pipe and the support are increased in the simulation [23,24]. Figure 3 shows the model diagram of the T-supported pipe, where (a) is the 3D model and (b) is the detection sectional drawing.

The defects are located on the centerline of the excitation sensor and the receiving sensor. The excitation signal of the sensor is a Hanning-window-modulated sine wave with an amplitude of 20 V and a number of cycles of five, and the receiving sensor is responsible for receiving the voltage signal, which is used to evaluate the detection performance of the CSH_0_ mode through the pipe support. Among them, the maximum cell size of the specimen is 1/6 of the wavelength. The time step is set to be less than 1/6 of the period, which can ensure the accuracy of the calculation results.

### 3.2. Simulation of the Support Structure

**Figure 4:**
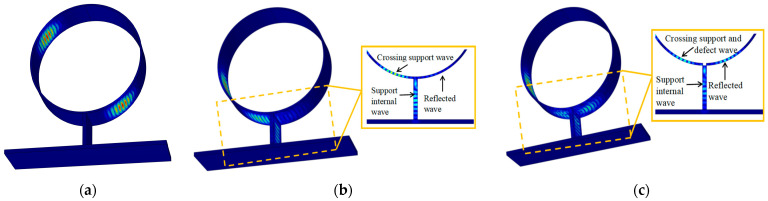
Sound field diagram at the support: (**a**) 50 μs; (**b**) 90 μs without defect; (**c**) 90 μs with defect.

The acoustic field of the PPM EMAT, with an excitation frequency of 500 kHz inside a 3 mm thick steel tube, is shown in Figure 4, where (a) is the observation curve of guided wave propagation before passing through the support at 50 μs; (b) and (c) are the observation curves of guided wave propagation after passing through the support at 90 μs; and there are pipe defects at the support in (c). A particle displacement cloud image can be used to observe the propagation characteristics of the CSH_0_ mode guided wave during and after its passing through the support.

**Figure 5:**
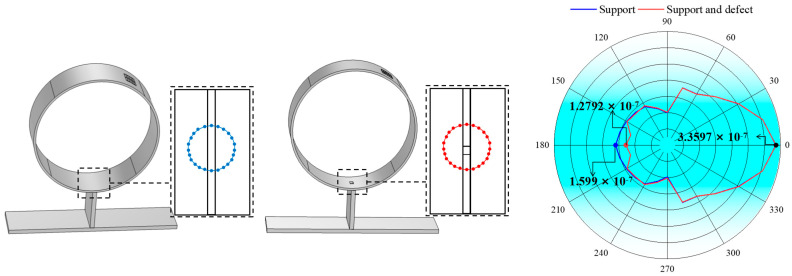
Displacement distribution before and after pipeline support structure.

In order to further extract the maximum displacement amplitude of the CSH_0_ wave before and after its passing through the support, the particle displacement amplitude model is obtained by taking the intersection point between the support position and the central axis of the sensor as the center, taking 20 mm as the radius, and receiving signals every 15°, as shown in Figure 5. The particle vibration displacement without passing through the support structure is 3.3597 × 10^−7^ mm. The guided wave passing through the support is divided into three parts: the reflected echo; the guided wave inside the support structure; and the guided wave passing through the support structure. When there is no pipe defect at the support, the particle displacement of the reflected echo of the support is 1.088 × 10^−7^ mm. The displacement of guided wave particles propagating to the interior of the support is 1.868 × 10^−7^ mm, and the displacement of guided wave particles passing through the support is 1.599 × 10^−7^ mm, accounting for 32.4%, 55.6%, and 47.5% of the amplitude of the other side, respectively. Among them, the backwave amplitude of the support reflection decreased by 67.6% compared to that before the cross-support.

Similarly, when there is a pipeline defect at the support, the particle displacement of the echo reflected from the support is 1.7025 × 10^−7^ mm. The displacement of guided wave particles propagating to the interior of the support is 1.5984 × 10^−7^ mm, and the displacement of guided wave particles passing through the support is 1.2792 × 10^−7^ mm, which are 50.7%, 47.6%, and 38.1% of the amplitude on the other side, respectively. The amplitude of the echo reflected by the support decreases by 49.3% relative to the amplitude before the cross-support.

The results show that the support structure not only reflects the guided wave but also weakens the amplitude of the detected signal. Therefore, the existence of support structures has a great impact on the detection of pipeline defects. When the pipe has defects, the guided wave amplitude reflected by the support increases by 18.3% compared to that without defects, indicating that the signal reflected by the support structure contains defect signals, and the pipeline defects at the support can be detected by this method.

**Figure 6:**
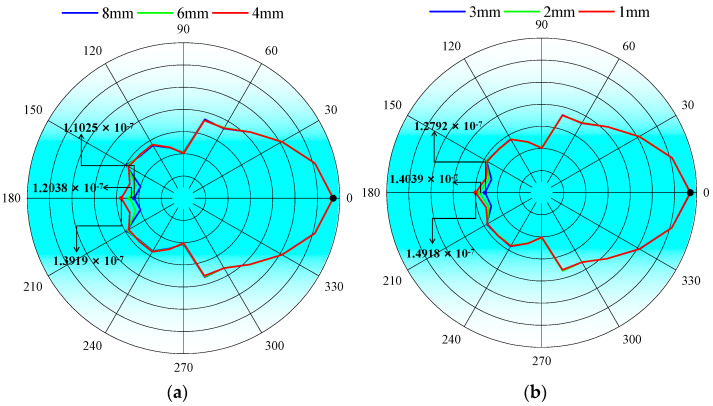
Comparison of particle displacement amplitude of pipe defects of different sizes before and after the support structure: (**a**) different lengths; (**b**) different depths.

When the defect length and defect depth are different, the attenuation degree of the signal after the guided wave crosses the support is also different. Figure 6a is a comparison diagram of the particle displacement amplitude of defects at different lengths of pipeline before and after the support structure, and Figure 6b is a comparison diagram of the particle displacement amplitude of defects at different depths of pipeline before and after the support structure. The results show that with an increase in defect length and depth, the attenuation of the guided wave across the support increases. It can be inferred that the amplitude of the reflected echo signal at the support structure increases with the increase in defect size.

## 4. Experimental System

**Figure 7:**
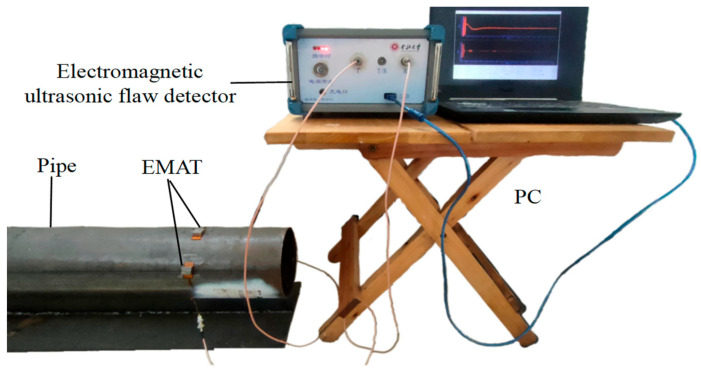
Diagram of the experimental system.

After the ability of the CSH_0_ guide wave to pass through the support structure was verified using a simulation, the experiments were designed according to the simulation steps. The experimental system based on the PPM EMAT mechanism was constructed. Figure 7 shows the schematic diagram of the experimental system, which mainly consists of a computer, an electromagnetic ultrasonic flaw detector (North University of China, Taiyuan, China), a PPM EMAT, and a steel tube with T-type support. The outer diameter is 180 mm. The thickness is 3 mm. The length is 1 m. The transducer, the periodic array magnet, and the runway-type coil correspond to the dimensional structure built into the simulation. The material of the periodic array magnet is NdFeB. The residual flux density is 1.4 T. The geometrical size is 10 mm × 3 mm × 10 mm (length × width × height). The runway-type coil is formed by using a double-layer PCB coil with an inner diameter and an outer diameter of 2 mm and 17 mm, respectively, and several turns of 30. The excitation sensor is 95 mm away from the receiving sensor, and the excitation sensor excites the CSH_0_ mode in the circumferential direction of the steel pipe. The generated signal is fed into the electromagnetic ultrasonic flaw detector through the computer, after which the receiving sensor receives the signal provided by the flaw detector after modulation. The electromagnetic ultrasonic flaw detector processes the data and finally feeds them back to the computer.

The support structure of the experimental pipeline is made of a footboard of the same material, with a length of 1 m, a width of 70 mm, and a thickness of 6 mm, and a base plate with a length of 1 m, a width of 250 mm, and a thickness of 6 mm, welded together.

**Figure 8:**
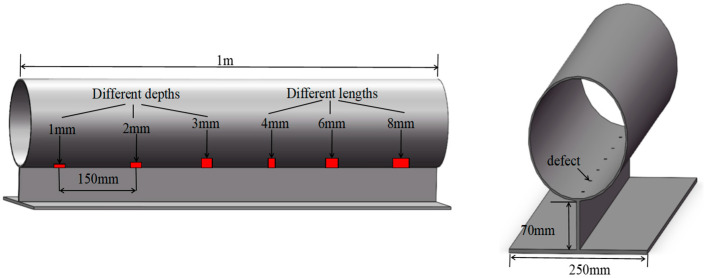
Schematic diagram of pipeline structure.

In this study, two steel pipes with the same structure were prepared, but their welding methods were different—one was spot welded, and the other was full welded—so as to explore the influence of the weld structure of the support on the SH_0_ wave. In the follow-up experiments, the interactions between SH_0_ waves and crack defects were studied. The defects are six square slots with a width of 5 mm. Three of them are through-square slots with sizes of 4 mm × 3 mm (length × depth), 6 mm × 3 mm (length × depth), and 8 mm × 3 mm (length × depth), and the others are non-through. The sizes are 5 mm × 1 mm (length × depth), 5 mm × 2 mm (length × depth), and 5 mm × 3 mm (length × depth). The axial distance of each defect is 150 mm. The structural diagram of its specific size and position is shown in Figure 8.

## 5. Results and Discussion

### 5.1. Influence of Pipeline Support Structure on SH Guided Wave

**Figure 9:**
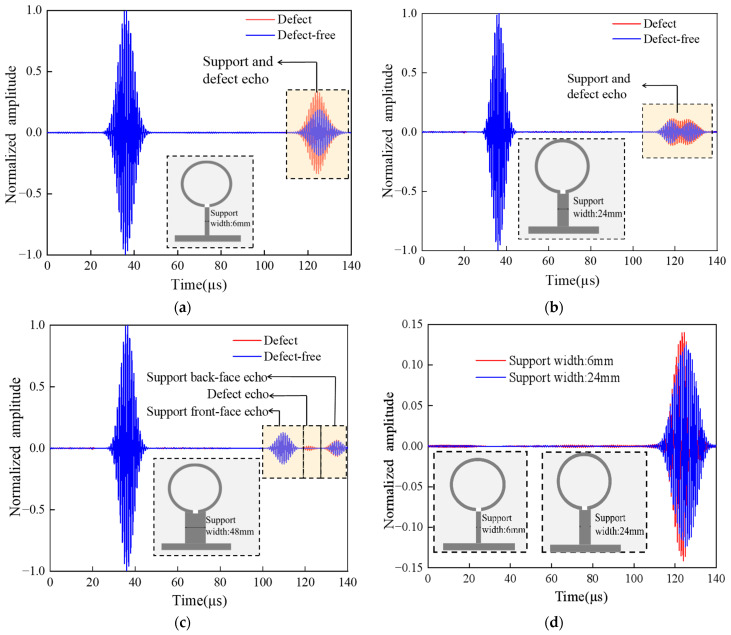
The signal receiving diagram of the support structure with different widths: (**a**) the support width is 6 mm; (**b**) the support width is 24 mm; (**c**) the support width is 48 mm; (**d**) the defect signal comparison diagram obtained by using waveform subtraction.

In order to study the influence of pipeline support structures on SH waves, three support structure models of different widths were set up, namely, 6 mm, 24 mm, and 48 mm. The influence of the three support plates on the pipeline defect signal was also studied. Figure 9a–c are the received signal diagrams of the support plates of these three widths. When the width of the support plate is 6 mm, the width of the pipe defect is 5 mm. When the pipeline is free of defects, there is only one wave packet at 110~130 μs, and the echoes of the front and back ends of the support plate overlap. When there is defect in the pipeline, the echo signal of the defect and the echo signal of the two ends of the support are superimposed, and the amplitude of the echo signal is obviously higher than that of the echo signal without a defect above the support. But it is very difficult to intuitively find defect information. When the width of the support plate is 24 mm, there are two incomplete separated wave packets at 110~130 μs, which are the echo signals of the front-end face and the back-end face of the support. When there is a pipeline defect above the support structure, there are three incomplete separated wave packets, the defect signal, and the echo signals of the two ends of the support. This makes the defect information more difficult to identify. When the width of the support plate is 48 mm, it can be seen that at 100~140 μs (Figure 9c), the front-end face and the back section echo are completely separated. When there is pipeline defect above the support, there are three obvious wave packets at 100~140 μs, namely, the front-end echo, the defect echo, and the back-end echo.

When the width of the support is between 6 and 24 mm, the defect echo is superimposed with the echoes of the two end faces of the support. In order to effectively extract the defect signal, waveform subtraction is used to process the data, and the defect signal diagram shown in Figure 9d is obtained. The results indicate that the smaller the width of the support plate, the greater the degree of signal superposition, and the more difficult it is to identify defect signals.

### 5.2. The Influence of Support Welding Quality on SH Wave

**Figure 10:**
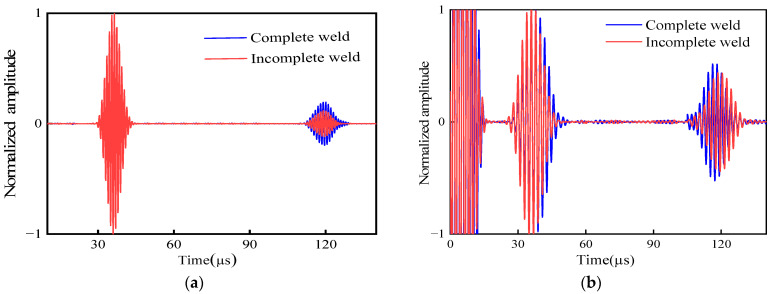
Signal detection diagram of pipe weld structure: (**a**) simulation results; (**b**) experimental results.

To verify the influence of the weld quality at the support structure on CSH_0_ wave propagation, an incomplete weld structure is compared with that of a complete weld structure at the support. Figure 10a shows the signal echo graphs with complete and incomplete welds in the simulation. The weld echo signal is in the range of 110~130 µs. In the experiment, the support structures of unwelded and fully welded pipes were selected for comparison. Figure 10b shows the experimental signal of fully welded and incompletely welded pipes and the weld echo signal is also in the range of 110~130 µs. It is consistent with the simulation results.

The echo signals of the two weld structures were normalized, and the peak echo signals of the weld were extracted. The results are shown in Table 1. In the simulation, the echo of an incomplete welding pipe attenuates by 30.2% compared with the return amplitude of a complete welding pipe. In the experiment, the echo of an incomplete welding pipe attenuates by 25.3% compared with the return amplitude of a complete welding pipe.

The results show that the reflection amplitude of the incomplete weld pipe is small, the influence of guided wave detection is small, and the defect signal of the channel can be easily detected. The echo signal amplitude is large when the weld structure is complete. A large part of the guided wave energy is reflected by the weld bracket when passing through the support structure, resulting in the attenuation and aliasing of the defect signal.

Therefore, when the pipe is fully welded, the defect detection difficulty is also increased. When the pipeline has defects, the echo signal includes the weld echo signal at the support and the pipeline defect signal at the support. In this study, a set of data, including the weld echo at the support of the pipeline and a set of superposition data of weld and defect echo signals, is obtained. If the excitation frequencies and amplitudes of the two columns of waves are the same, the absolute value of each point of the two columns of waves is taken, the required signal value is obtained through subtraction operations, and Gaussian filtering is carried out to make the waveform trimmed and smoother. So, to extract the pipeline defect signal at the support, waveform subtraction was used to eliminate the influence of the weld echo and then effectively extract pipeline defect signals.

**Figure 11:**
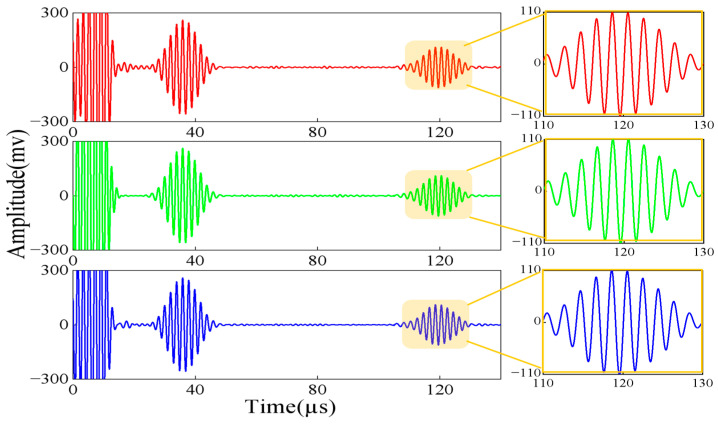
Signal detection of T-type pipe without defect under full welding condition.

In the follow-up experiment, the fully welded pipe was selected for further study. The experimentally received defect-free signal at the support is given in Figure 11. The amplitude of the weld echo at the support is stabilized at about 110 mv for several measurements. Therefore, we can choose one of the sets of data as the value to be subtracted in the waveform subtraction for subsequent data processing.

### 5.3. Study of Defects of Different Length Sizes

In this study, the selected sensor combination is a racetrack coil and a periodic array magnet to generate a CSH_0_ wave, and the relationship between its excitation frequency, phase velocity, and wavelength is as follows:

(2) $$d=\frac{\lambda }{2},\lambda =\frac{Cp}{f},$$

where *d* is the width of the periodic array magnet, *Cp* is the phase velocity, *f* is the excitation frequency, and *λ* is the wavelength.

**Figure 12:**
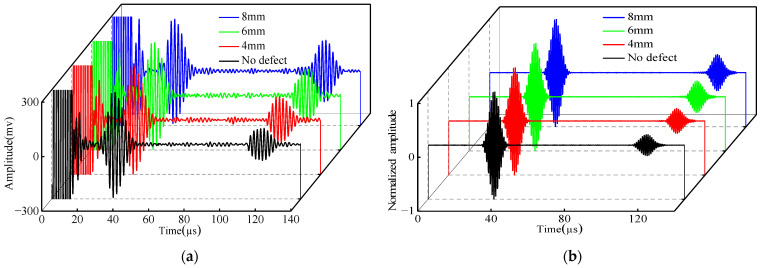
Comparison of experimental and simulated signals for different length defects at the support: (**a**) experimental; (**b**) simulated.

Figure 12 gives the experimental simulation normalized signals of no defects at the support and three types of crack length defects at the support. The comparison shows that there are two wave packets that clearly existed in both the simulation and the experiment, which are the direct wave (wave packet I) and the welds at the support and the defects return wave (wave packet II). According to the relative position of the defects and the transducer in the experiment, the propagation distance is 378 mm. According to the dispersion curve in Figure 2, the group velocity of the CSH_0_ wave is 3142 m/s. After calculation, the time to receive the defective echoes should be around 120 μs. Obviously, wave packet II is the echo of defects and welds. The echo time can be seen in 110~130 μs according to the signal diagram, which is the same as the calculation results. Similarly, in the simulation, wave packet II appears between 110 and 130 µs; that is, it is the echo of weld and defect, which is consistent with the experimental results.

**Figure 13:**
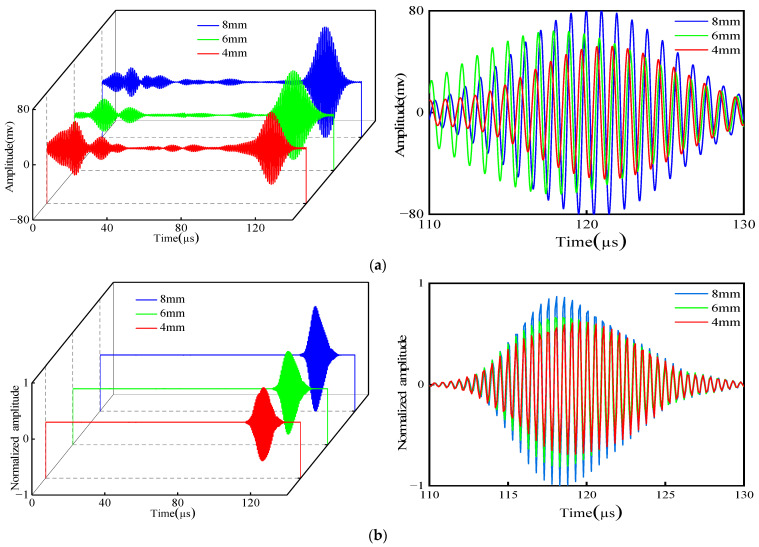
Comparison of experiment and simulated defect signal after waveform subtraction: (**a**) experiment; (**b**) simulation.

To find the defect signal hidden in echo II, it is necessary to eliminate the influence of the support weld. Therefore, the signal is processed using the waveform subtraction method. Three groups of defect signals are obtained, respectively. An enlarged view of the defect signal is plotted below the 3D plot group in Figure 13.

**Figure 14:**
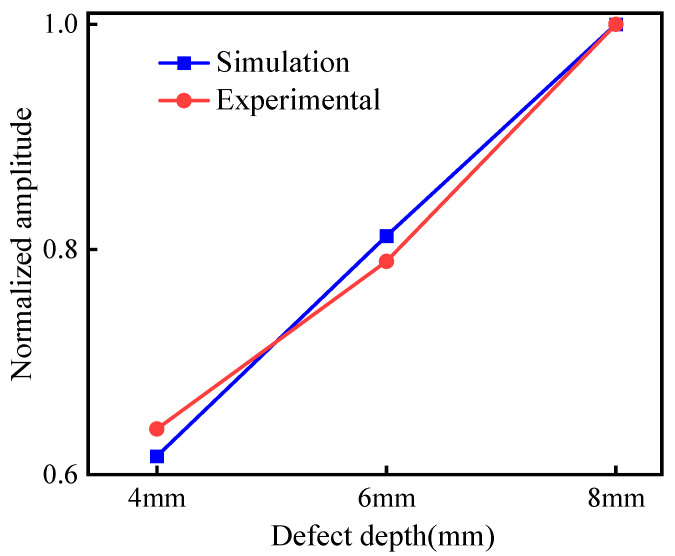
Comparison of different lengths.

Subsequently, the peak signals were extracted and normalized, and the results are shown in Table 2. The plotted amplitude comparison chart is shown in Figure 14. The simulation and experiment have the same law. When the defect size increases, the detection amplitude of defects at the support also increases, indicating that the method can effectively detect the defects existing at the support. When the size of the defects decreases, the sensitivity of detection also decreases.

### 5.4. Study of Defects at Different Depths

**Figure 15:**
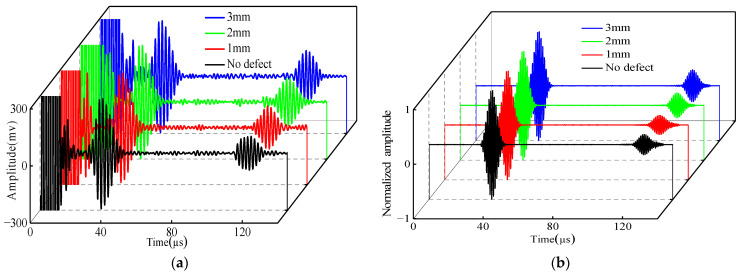
Comparison of different depths: (**a**) experimental; (**b**) simulated.

Figure 15 gives the normalized signals of the experimental simulation with no defects at the support and three different depths of defects at the support. The comparison shows that there are two wave packets that existed in both the simulation and the experiment, i.e., the direct wave (wave packet I) and the weld and defect echo wave (wave packet II) at the support.

**Figure 16:**
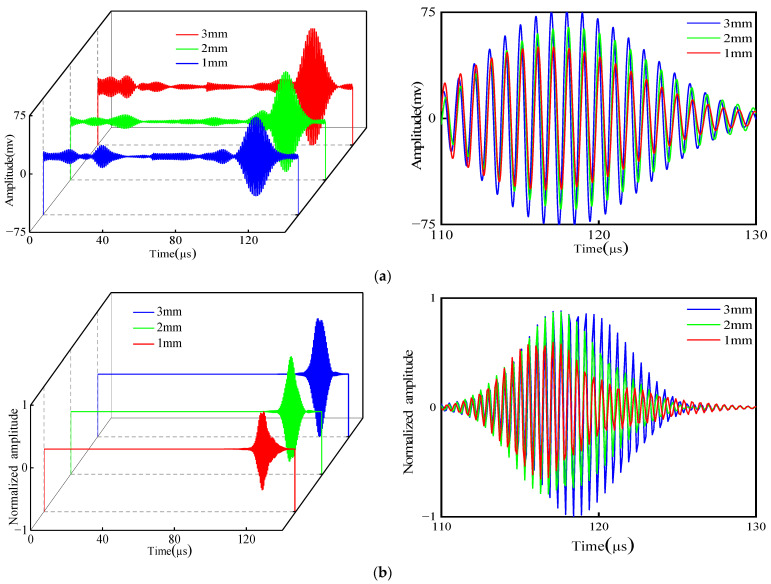
Comparison of experimental and simulated defective signals after using base reduction method: (**a**) experimental; (**b**) simulated.

Similarly, to eliminate the echo signals at the support weld, the signals are processed using the waveform subtraction method. Three groups of defect signals are obtained. The enlarged diagrams of the defect signals are plotted on the right side of Figure 16.

**Figure 17:**
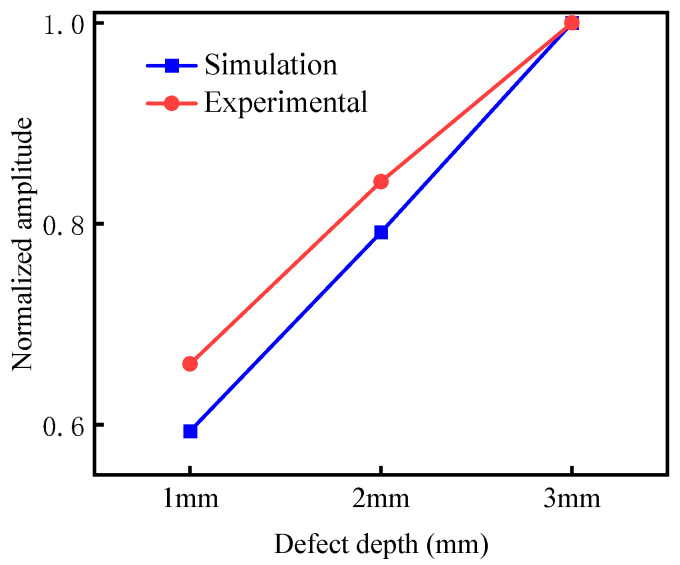
Comparison of different depths.

Peak signals were extracted and normalized, as shown in Table 3 and Figure 17. According to the graph, it can be seen that the simulation and experiment have the same law. The deeper the defect, the more amplitude of the detection of defects at the support increases. When the depth of the defect decreases, the sensitivity of the detection decreases too. Compared with defect signals of different lengths, the amplitude and sensitivity of the defect signals at different depths are smaller. Further studies on the defects at different depths are needed.

## 6. Conclusions

A waveform subtraction method with circumferential shear and horizontal guided waves is proposed, which is generated by an electromagnetic acoustic transducer. The conclusions are as follows:

(1) By analyzing the cloud image of the pipeline, the results show that when the pipeline defect is above the T-type support structure, the amplitude of the reflected echo at the support increases by 18.3% compared with that when the pipeline has no defect, so the reflected echo of the support contains defect signals. Therefore, this method can effectively detect the defect.
(2) The simulation results show that the different widths of the support structure have different effects on the guided wave signal. The results indicate that the smaller the width of the support plate, the greater the degree of signal superposition, and the more difficult it is to identify defect signals. The experimental and simulation results show that the reflection amplitude of pipes with good welding quality is large, and defect detection is more difficult than that of pipes with poor welding quality. In addition, the pipeline defect signal is proportional to the length and depth in the defect. With the decrease in defect size and depth, the detection sensitivity also decreases.
(3) This method can effectively detect a 5 mm × 1 mm (length × depth) non-through crack hidden above the support structure. These results provide the basis for the research on the detection of large defects in the pipeline with the existence of the T-type support structure. However, when the defect size is smaller or the depth is smaller, the influence of the weld structure impedes the detection of the defect. Future study needs to focus on optimizing for small defect detection.

## Figures and Tables

**Table 1:** Normalized amplitude of defect echoes for different weld structures.

Weld Structure	Complete Weld	Incomplete Weld
Simulated signal amplitude	0.195	0.136
Experimental signal amplitude	0.514	0.384

**Table 2:** Simulated and experimental normalized amplitudes of defective echoes of different lengths.

Crack Defect Size (mm)	4	6	8
Simulated defect amplitude	0.617	0.812	1.000
Experimental defect amplitude	0.641	0.790	1.000

**Table 3:** Simulated and experimental normalized amplitudes of defective echoes of different depths.

Defect Depth (mm)	1	2	3
Simulated defect amplitude	0.586	0.790	1.000
Experimental defect amplitude	0.641	0.834	1.000

## Data Availability

The original contributions presented in the study are included in the article, further inquiries can be directed to the corresponding author.
